# Tooling nurses to assess complexity in routine home care practice: Derivation of a complexity index from the interRAI‐HC

**DOI:** 10.1002/nop2.686

**Published:** 2020-11-25

**Authors:** Catherine Busnel, Fanny Vallet, Catherine Ludwig

**Affiliations:** ^1^ Research and Development Unit Geneva Institution for Homecare and Assistance (imad) Carouge Switzerland; ^2^ Geneva School of Health Sciences, HES‐SO University of Applied Sciences and Arts Western Switzerland Geneva Switzerland

**Keywords:** assessment, complexity, home care, interRAI‐HC, nurses, nursing

## Abstract

**Aim:**

Home care nurses often use the Resident Assessment Instrument‐Home Care (interRAI‐HC) to assess health needs. However, this tool does not assess complexity. This study proposes to derive a complexity index (CI) from the interRAI‐HC using the operational definition of the dedicated COMID checklist (COmplexité Multidimensionnelle des prises en soins Infirmières à Domicile).

**Design:**

Data were collected at the baseline assessment of the fraXity study (*N* = 231, aged ≥ 65), which relied on an observational longitudinal design.

**Methods:**

Measures were the interRAI‐HC, from which the CI binary variables were computed and the COMID, used as a reference.

**Results:**

Twenty‐six CI variables were computed from the interRAI‐HC, and all but three correlations were significant. The correlation between the CI score and the COMID score was *ρ* = 0.730 (*p* < .001).

**Conclusions:**

The study demonstrates that complexity can be assessed directly from the interRAI‐HC by deriving a CI.

## 1 INTRODUCTION

In Switzerland and more particularly in the canton of Geneva, home care has a central place in the delivery of curative, preventive, educational and palliative care. The organization of home care is a response to the population's desire to stay at home for as long as possible and to receive home assistance and care services. With the ageing population, home care nurses are increasingly faced with patients with multiple clinical, chronic and fluctuating conditions (Valderas et al., 2009), who are at high risk of decompensation and hospital readmission (Joyce et al., 1981; Koné Pefoyo et al., 2015). These situations are alternatively characterized by “patient complexity” (Peek et al., 2009), “case complexity” (de Jonge et al., 2005), “care complexity,” “needs complexity” (de Jonge et al., 2006) or “practice complexity” (Davidson et al., 2011), suggesting that complexity is a multifaceted construct. Complexity can be broadly defined as a “multidimensional concept involving interactions between biological, socioeconomic, cultural, environmental and behavioral forces as health determinants” (Bonizzoni et al., 2018). In the same vein, the World Health Organization (WHO, 2009) posits that “a complex system is one where there are so many interacting parts that it is difficult, if not impossible, to predict the behavior of the system based on a knowledge of its component parts.” The term “complex situation” is often used by professionals in their practice and although it may cover heterogeneous realities and definitions, it consensually refers to the features of non‐linear and dynamic systems (Plsek & Greenhalgh, 2001). Considering a biopsychosocial perspective (Engel, 1980), not only does the presence of one or multiple chronic diseases contribute to rendering situations complex, but also social and psychological dimensions. Contributing to this complexity is also the characteristics of the care, especially linked with the presence of multiple formal or informal actors who interact with each other and with the healthcare system, as well as the instability of the situation (Shippee et al., 2012). Complexity in care has developed either through the biomedical approach or through the determinants of health approach (vector model of complexity; (Safford et al., 2007)). In the home, these two approaches are important and need to be considered together (Johnson & Bacsu, 2018). Home care requires nurses to take a multidimensional, interdisciplinary and holistic approach. They must take into account factors intrinsic to the patient (health, religion, socioeconomic status), factors related to health professionals (physicians, nurses, others), factors related to the delivery of care, organizational factors, the team environment (structure, planning) and political factors. The nurse must mobilize in his or her practice “complex thinking,” “complexity assessment” (assessment of multidimensionality) and “complex acting” (interdisciplinarity and interprofessionality) (Richard et al., 2012). Complexity is a construct that concerns the person, the environment, health and care, and as such, complexity can be understood in the light of the nursing metaparadigm (Busnel et al., 2020; Fawcett, 1984).

To face the challenges of complexity in daily nursing practice, some models have been developed, such as the chronic care model (Bodenheimer et al., 2002), to anticipate and coordinate care management and to avoid therapeutic incoherence, which is resource‐consuming. Some tools have been developed to assess complexity, but they are mostly designed for use by physicians (Huyse et al., 1999) in hospital settings (Stiefel et al., 2006). In the current care practice less‐centred on the hospital model and more on ambulatory and domiciliary care, few instruments enable evaluating complexity. To our knowledge, the only instrument available for home care nurses is a multidimensional complexity assessment instrument, known as the COMID (abbreviation for the French locution “COmplexité Multidimensionnelle des prises en soins Infirmières à Domicile,” or in English, Multidimensional Complexity Assessment Instrument for Home Nursing Practice) (Busnel et al., 2018). Currently, nurses are using the COMID as a complement to the routine comprehensive health assessment. Specifically, the COMID consists of a 30‐item checklist coding for the presence or absence of characteristics of “case complexity” (medical circumstances, socioeconomic circumstances, aggravating mental circumstances and aggravating behaviour), “care complexity” (circumstances of care delivery) and “instability.” Home care nurses who intervene regularly at patients' homes are in the primary position to assess the whole situation of the patients and their needs with regard to the context to plan and coordinate the care. Given the amount of information the nurse has to consider when establishing an intervention plan, some instruments were developed in clinical practice to assess the needs of the patient and to support clinical reasoning. One of these standardized instruments is the Resident Assessment Instrument‐Home Care (interRAI‐HC), widely used to evaluate the needs of the patients requiring care at home in various domains of health (e.g. pain and behaviour). The interRAI‐HC is an instrument dedicated to comprehensive geriatric assessment. The RAI‐HC is used by nurses to evaluate each new patient's care and to regularly re‐evaluate patients in long‐term care. To structure this data collection, the minimal data set (MDS) of the interRAI‐HC is accompanied by alerts and scales targeting various health conditions (Morris et al., 1999). Interestingly to the present purpose, the interRAI‐HC does not offer a specific indicator/alert on complexity. These alerts and scales serve to support clinical reasoning in alerting the nurse about a potential risk in a given domain and in guiding the nurse's analysis to make decisions about further investigation requirements or care needs.

In addition to these implemented alerts and scales, research on relevant concepts in the domains of gerontology and/or of care (Armstrong et al., 2010; Morris et al., 2016) has developed the derivation of other clinical indicators or indexes, with the aim of saving time and avoiding the use of additional external scales. Such indexes give extra information to the nurse, who analyses it and may adapt the care plan accordingly. Among the proposed indexes, a frailty score is gaining popularity (Hubbard et al., 2015; Ludwig & Busnel, 2017, 2020; Searle et al., 2008). The purpose of developing the frailty index was to facilitate the detection of frail patients and to foster a careful analysis of their needs to prevent functional decline. The derivation of an index aims at structuring the data collection in the clinical context to help nurses in their routine assessments. The role of home care nurses to provide care in a coordinated and meaningful way can increasingly be challenged by the presence and interactions of multiple factors, resulting in a complex pattern of patients, patients' needs and care that may render the clinical analysis of the situation difficult. Therefore, nurses need to be equipped with instruments supporting the detection, synthesis and analysis of the situational elements that contribute to complexity. However, complexity is rarely operationalized and few tools are adapted to the home care nursing context (e.g. the COMID) (Busnel et al., 2018; Vallet et al., 2019). The aim of this study was to propose a computation algorithm to derive a complexity index (CI) from the interRAI‐HC that complies with the operational definition of complexity provided by the COMID. The value of the proposed approach is to provide nurses with a complexity score directly available in routine assessments (with the interRAI‐HC), hence fostering coherence and saving time (avoiding the need to use an additional COMID assessment). Explicitly stated, the research question addressed by the study is “Can the interRAI‐HC be used to derive a complexity index (CI) that complies with the operational definition of complexity provided by the COMID?” Answering this question first implies identifying a set of interRAI‐HC items that allow for creating scores that mirror the content of the COMID and second, to test the proposed values against the corresponding ones obtained with the COMID.

## 2 METHODS

### 2.1 Design

The data used to derive the CI from the interRAI‐HC and to validate the score against the COMID were collected at the baseline assessment of the fraXity study (Ludwig & Busnel, 2019) from 30 October 2018–12 May 2019. fraXity is an observational longitudinal study; its protocol is extensively described elsewhere (Ludwig & Busnel, 2019).

### 2.2 Setting and sample

A sample of 231 individuals aged 65 years or older living in the community were enrolled in the fraXity study (Ludwig & Busnel, 2019). Participants lived in private dwellings in the canton of Geneva, Switzerland. They were fluent in French and free of major cognitive or communication deficits. All participants volunteered and gave written informed consent for participation. From the fraXity sample, 216 participants (mean_age_ = 79.35, *SD* 8.1, 78.2% females) were considered for the present analysis.

### 2.3 Data sources and measurement

Data were collected through interviews at the participants' homes in conditions as close as possible to real clinical conditions. Nurses were trained in the use of the instruments included in the protocol. Among other measures, the instruments included the interRAI‐HC and the COMID, collected during a single interview conducted by a nurse.

Data used to derive the CI were collected from the French Canadian interRAI‐HC (edition v.9.1) (Morris et al., 2009) as used in the fraXity study. The instrument belongs to the RAI instrument suite developed by the interRAI consortium (https://www.interrai.org/). The interRAI‐HC is designed as a tool guiding comprehensive geriatric assessment and is available in many languages. All these national/local versions rely on a common structure and a common item coding system to foster coherence and comparability. The interRAI‐HC covers 19 health‐related domains: (A) administrative information; (B) living conditions; (C) cognition; (D) sensory abilities; (E) health‐related behaviours; (F) social behaviours; (G) activities of daily living; (H) continence; (I) medical diagnoses; (J) falls, physical abilities, physical symptoms and pain; (K) nutrition; (L) skin and feet problems; (M) medication; (N) ongoing therapies and formal care; (O) advanced care instructions and legal representation; (P) informal care; (Q) living environment; (R) observed change in activities of daily living; and (S) record information. The instrument embeds 24 clinical assessment protocols and 18 clinical scales. In the clinical setting of the study, the interRAI‐HC is routinely used by nurses to evaluate the care needs of each home care patient at admission and in routine reassessments.

Another instrument analysed in the present study was the COMID, which was considered a standard from which to choose the different interRAI‐HC items to compose the CI variables. The COMID is an instrument for assessing multidimensional complexity in home care nursing practice and is completed by home care nurses in addition to a comprehensive health assessment to support their clinical analysis of complexity. Based on factors identified in the literature, the COMID was developed to provide an operational definition of complexity by identifying variables that contribute to the complexity of home care situations. It is a checklist of 30 binary items, with 5 items in each of the 6 complexity domains (medical health factors, social and economic factors, mental health factors, behavioural factors, instability factors and factors related to care providers and the care system). The COMID, developed in French (an English version is also available at https://comid.imad‐ge.ch/), has shown good acceptability (Busnel et al., 2018) and reliability (Vallet et al., 2019). Based on their clinical assessment of a given situation, nurses code 1 (“yes”) if the item is present or 0 (“no”) if it is absent. In its original version, the total COMID score is calculated by summing the “yes” responses over the 30 items (COMID‐30) and can range from 0–30, with a higher score indicating a greater accumulation of factors contributing to the complexity of a situation.

### 2.4 The complexity index variables derived from the interRAI‐HC

The principle used for creating the complexity index (CI) was to first identify variables in the interRAI‐HC that mirror the COMID items. For each COMID item, one or more interRAI‐HC items were combined to find the best proxy. Each of the interRAI‐HC candidate items was used only once. Some CI variables were composed of single interRAI‐HC items when they were similar or very close to the formulations used in the COMID variable (e.g. financial difficulties). For other CI variables, a combination of several items fit the definition of the COMID variable. For instance, the CI variable 2d, a situation where patients live alone and have few social interactions or who report a change in social activities, fits the definition of social isolation. The choice of the interRAI‐HC items used to compute the CI variables, as well as their best combination to mirror the COMID, relied on a consensus‐reaching approach involving two clinical experts. Ultimately, each variable composing the CI was binary. In case of divergent opinions across experts, phi tests were conducted to assess the link between a given CI variable and its corresponding COMID variable. The combination with the highest coefficient value was selected.

A total of 26 CI variables were created, and four variables of the COMID items, linked with the care providers and the care system dimension, could not be derived from the interRAI‐HC. These items were 6b, absence or low degree of partnership between the different actors; 6c, therapeutic incoherence; 6d, health insurance problems; and 6e, emotional and/or physical burden perceived by the secondary network.

Table 1 presents the selected computation used to create each of the 26 CI variables. As the version of the interRAI‐HC used in fraXity is comparable to the Standard English edition v.9.1.2, the coding used to create the CI was employed for the standard version to be used by as many people as possible (all details about the formula and the specificity of coding in fraXity are presented in Table S1). The total complexity score was calculated by summing the 26 variables composing the CI, for a total score ranging from 0–26.

**TABLE 1:** Correspondence between the COMID items and interRAI‐HC items used for the derivation of the Complexity Index

COMID		interRAI‐HC	
Item	Var. label	Item	Var. description
1a	Several chronic diseases (more than 2) and/or unexplained symptoms	I1c, I1d, I1f, I1h, I1j, I1k, I1l, I1m, I1t, I1u, BMI (K1b, K1a), J3u, J4, I2	More than 2 chronic diseases among a list of 13 diseases (plus one disease counted for any response in the question “other diagnostic”)
1b	Chronic pain	J6a	Any pain
1c	Any allergies and/or drug intolerances	M2	A known drug allergy
1d	Polymedication	M1f (M2f, M3f, M4f, M5f, etc.)	Five or more substances regularly taken
1e	Cognitive deficits	C2a, C1, C2b, C2c	Short‐term memory problem and at least one another deficit among the following cognitive functions: decision‐making, procedural memory or long‐term memory
2a	Financial difficulties and/or an inability to afford the services of assistance, care, treatments, auxiliary devices, a means of transportation and/or a food supply	Q4	Financial difficulties
2b	No informal care, an exhausted informal caregiver and/or family tensions	P1a1, P1a2, P4, P2a, P2b, P2c	No informal caregiver/helper and no supportive relationship with family OR a caregiver/helper who is unable to continue his/her help or reports distress/anger or is overwhelmed
2c	Low level of literacy (related to alphabetization issues, language and/or cultural barriers)	D2	Not good and clear understanding
2d	Social isolation	A13a, F1b, F1c, F3	Living alone and: not visiting or receiving visits from family and friends and not having other interactions during the last 3 days, or a change in social activities
2e	Inadequate housing and/or environmental barriers	Q1a, Q1b, Q1e	Any problem with (or uncertainty about) degradation, squalid conditions or limited access to housing
3a	Depression and/or suicidal ideation	I1p, E2c	Diagnostic of depression or self‐reported depressed mood
3b	Psychiatric diseases and/or mental disorders (delusions, hallucinations, etc.)	J3g, J3h, J3i, I1q, I1o	Any symptoms of abnormal thought process, delusions or hallucinations, or a diagnosis of psychosis or bipolar disorder
3c	Addiction	J9a, J9b	Daily smoking or consumption of at least 5 drinks of alcohol in one go
3d	Anxiety or anguish that renders the clinical picture unclear	I1n, E2b	Diagnostic of anxiety or self‐reported anxious mood
3e	Variations in mental function during the day	C3c	Mental function varies over the day
4a	Recurring solicitations of the primary and/or secondary network	E1e	Repetitive anxious complaints
4b	Ambivalent and/or conflictual communication with a member of the primary and/or secondary network	F1d, E1b	Conflicts/angry with friends or family and a perpetual anger against oneself or others
4c	Worries about symptoms, health conditions, and/or medical information	E1d	Repetitive health complaints
4d	Aggressiveness (verbal and/or physical) or mutism	E3b, E3c	Any manifestation of verbal or physical aggressiveness
4e	Resistance or opposition to care, whether active or passive	E3f	Manifestation of resistance to care
5a	Recent degradation of health status perceived by the patient	J7b	Experiencing an acute crisis or flare‐up of a recurrent or chronic problem
5b	Overall change in the degree of independence (ADL/IADL) in the last month	G6, R2	Deterioration (or uncertainty) of the ADL performance or a significant change in general independence
5c	Transition period (ex. announcement of diagnosis, hospital discharge, death of caregiver, divorce, work, etc.)	F5, A14, A13b	Major life stressor or hospitalization or change in the household composition
5d	Acute change in cognitive abilities	C5	Deterioration (or uncertainty) of the decision‐taking capacities during the last 3 months
5e	Unpredictability of health status (unusual symptoms, decompensation of a chronic disease, wounds, pain, etc.)	J7a, J7c, C4, N4b	Health conditions or diseases making cognitive state, ADL, mood or behaviour patterns unstable or end‐stage disease or acute change in the mental state with regard to the usual functioning, or at least two visits to the hospital emergency
6a	Multiple care providers in the secondary network (primary care doctors, medical specialists, formal caregivers, curators, etc.)	N3aA, N3bA, N3cA, N3dA, N3eA, N3fA, N3gA, N3hA, O1	At least three providers

Abbreviations: ADL, activities for daily living; IADL, instrumental activities for daily living.

### 2.5 Data analyses

Beyond the descriptive analyses presenting the distribution of frequency of responses for each CI variable (and comparatively for the COMID variables), phi tests were conducted to test the relationship between each of the 26 CI variables and its corresponding COMID variable. Given the number of phi tests that were conducted to assess the relationship between the CI variables and the COMID variables, the 5% risk of type I errors needed to be adjusted for multiple comparisons. Bonferroni's correction was applied to adjust the *p*‐value to the *α* = 0.05 threshold used to reject the null hypothesis. An adjusted *p*‐value of .0019 was used (*α*/26, the number of CI variables). The internal consistency of the CI was tested with Cronbach's alpha.

The correlation between the CI total score and the COMID total score was assessed by means of Spearman's rank correlation coefficient. To be comparable with the CI total score, the total score of the COMID was computed by summing the “yes” responses on the 26 variables (COMID‐26) from those used as a reference for the creation of the CI. It should be noted that because the number of “yes” answers for the four excluded COMID items was low (i.e. it did not exceed 7, being 3.2%, for the item 6e, emotional and/or physical burden perceived by the secondary network), this should not have a strong impact on the total score.

Missing data on the relevant variables considered to compute the CI variables and their correlations with the COMID (i.e. any missing data on the CI variables or on the COMID‐30 variables: *N* = 15, representing 6.5% of the whole sample) were not replaced. Analyses were conducted using the list‐wise deletion method.

### 2.6 Ethics

The study protocol was approved by the Ethics Committee of the canton of Geneva, Switzerland (affiliated with 253 Swissethics) on 7 August 2018 (registration number: 2018‐01039). The study protocol was a prospective observational design using coded data on non‐genetic personal health data.

## 3 RESULTS

### 3.1 Participants

From the fraXity sample of 231 participants, only those with full data on every item of the CI and the COMID were retained for the analyses (*N* = 216). This final sample was aged from 65–97 (mean_age_ = 79.35, *SD* 8.1), with 78.2% females.

### 3.2 Complexity Index

#### 3.2.1 CI variables: description and comparison with COMID variables

Descriptive analyses for each of the CI variables and its corresponding COMID variable are presented in Table 2. The number of “yes” responses (i.e. element coded as problematic) was different between the CI variables. Some variables were frequently rated “yes” (e.g. 1b, chronic pain: *N* = 155 “yes”), and others were rarely or never rated “yes” (e.g. 4e, resistance or opposition to care: *N* = 0 “yes”). The number of “yes” responses was relatively similar between the CI and the COMID for several variables, with some exceptions (e.g. 1a, chronic diseases).

**TABLE 2:** Number of “yes” answers for the COMID items, the corresponding values for the CI and the results of the phi test (coefficient, *p*‐value) assessing the correlation between each pair

Short title of the variable	COMID		CI		Correlation (phi, *p*‐value)
	No	Yes	No	Yes	
1a. Chronic diseases	110	106	140	76	0.52, *p* < .001
1b. Chronic pain	73	143	61	155	0.84, *p* < .001
1c. Allergies/drug intolerances	162	54	159	57	0.89, *p* < .001
1d. Polymedication	131	85	126	90	0.82, *p* < .001
1e. Cognitive deficits	197	19	168	48	0.38, *p* < .001
2a. Financial difficulties	204	12	193	23	0.64, *p* < .001
2b. Absence or exhaustion of informal caregiver	195	21	167	49	0.38, *p* < .001
2c. Low level of literacy	208	8	197	19	0.29, *p* < .001
2d. Social isolation	191	25	186	30	0.32, *p* < .001
2e. Inadequate housing	193	23	195	21	0.44, *p* < .001
3a. Depression and/or suicidal ideation	198	18	167	49	0.48, *p* < .001
3b. Psychiatric diseases	212	4	208	8	0.34, *p* < .001
3c. Addiction	202	14	197	19	0.52, *p* < .001
3d. Anxiety or anguish	200	16	155	61	0.37, *p* < .001
3e. Mental function varies over the day	209	7	210	6	0.45, *p* < .001
4a. Recurring solicitations	206	10	190	26	−0.01, *p* = .839
4b. Ambivalent and/or conflictual communication	214	2	211	5	0.31, *p* < .001
4c. Worries about symptoms	193	23	174	42	0.29, *p* < .001
4d. Aggressiveness	214	2	212	4	0.35, *p* < .001
4e. Resistance or opposition to care	214	2	216	0	Not calculated
5a. Recent degradation of health status perceived by the patient	179	37	199	17	0.28, *p* < .001
5b. Change in the ADL/IADL	201	15	184	32	0.40, *p* < .001
5c. Transition period	194	22	149	67	0.20, *p* = .003
5d. Acute change in cognitive abilities	210	6	205	11	0.35, *p* < .001
5e. Unpredictability of health status	180	36	186	30	0.47, *p* < .001
6a. Multiple care providers	205	11	190	26	0.37, *p* < .001
Original COMID items					
6b. Absence or low degree of partnership between the different actors	214	2			
6c. Therapeutic incoherence	216	0			
6d. Health insurance problems	214	2			
6e. Emotional and/or physical burden perceived by the secondary network	209	7			

Abbreviations: ADL, activities for daily living; IADL, instrumental activities for daily living.

The phi tests (Table 2) showed that 23 CI variables correlated significantly with the corresponding COMID variables, and the phi test for one variable (i.e. 4e, resistance or opposition to care) was not calculated because the number of “yes” responses on the CI was 0. The highest values for phi tests (>0.50) were obtained for 1a, chronic diseases; 1b, chronic pain; 1c, allergies/drug intolerances; 1d, polymedication; 2a, financial difficulties; and 3c, addiction. The lowest and non‐significant values (≤0 0.20) were obtained for 4a, recurring solicitations, and 5c, transition period.

Complementary results about the number of “yes” responses and phi tests obtained for the whole sample using all the available data for each analysis (i.e. pairwise deletion method) are presented in the Table S2. Globally, these results presented no major differences with the pairwise deletion method, as the values of the phi tests were largely comparable.

#### 3.2.2 Internal consistency

The CI exhibited an acceptable internal consistency (Cronbach's alpha = 0.689), comparable with that of the COMID‐26 (Cronbach's alpha = 0.763) and the original version of the COMID (COMID‐30, Cronbach's alpha = 0.770).

#### 3.2.3 CI total score: description and comparison with the COMID total score

Regarding the total scores, the CI total score had a mean = 4.49 (*SD* 3.04, Min = 0, Max = 14), which was higher than the mean of the COMID‐26 = 3.34 (*SD* 2.94, Min = 0, Max = 17). This suggests that the CI (just like several CI variables) was more sensitive than the COMID.

The correlation between the CI and COMID‐26 was significant, with Spearman's rank coefficient correlation of *ρ* = 0.730, *p* < .001.

## 4 DISCUSSION

### 4.1 Derivation of a CI

This aim of the study was to derive a CI from the interRAI‐HC based on the operational definition of complexity from the literature used to create the COMID. After a careful selection of interRAI‐HC items, 26 CI variables were computed—using different methods of computation—to match 26 out of the 30 COMID binary variables. When the correspondence between the CI variables and the COMID ones was assessed, the results were satisfying. The correlation tests were significant for 23 variables, and the phi values were substantial. For the two non‐significant phi values (i.e. 4a, recurring solicitations, and 5c, transition period) and the one correlation that could not be calculated (i.e. 4e, resistance or opposition to care), further testing on other samples would be needed and some adjustments might be necessary. Nevertheless, taken together, correlational analyses support the combinations chosen to create the CI variables. Otherwise, the internal consistency of the CI was high (i.e. 0.689) and similar (i.e. <0.1 difference) to that of the COMID. The total score of the CI, corresponding to the sum of the CI variables, was strongly and significantly correlated with the COMID‐26 total score. These results mean that the CI total score presented an acceptable reliability similar to that of the COMID and shares a large part of its variance with the checklist. Overall, the results of this study support the possibility to create a CI based on 26 variables that complies with the operational definition of complexity of the COMID. The variables and the CI score demonstrate suitable characteristics (i.e. internal consistency comparable with that of the COMID, a large number of significant correlations), and as a whole, the study can be viewed as a proof of concept supporting the derivation of a CI from the interRAI‐HC MDS.

### 4.2 Clinical application

The creation of a CI follows the creation of other indexes from the interRAI‐HC, offering a new index assessing complexity but different conceptually and in its computation from other indexes derived from interRAI instruments. As complexity is often conceptualized but rarely measured, creating a CI from the interRAI‐HC is a unique opportunity to assess complexity based on existing and widely used instruments.

In clinical practice, the possibility to create the CI variables automatically should help nurses to detect complex situations and to orientate the assessment and analysis of the elements contributing to the complexity. To enable routine use, it will be necessary to develop the CI as an alert by complementing the algorithm with clinical assessment protocols. When the CI variables alert the nurses about the risk of complexity of a situation, it would be possible to complement this by filling in a tool, such as the COMID. This would enable evaluation of the nurse's clinical analysis of the situation and deepen the assessment of care system complexity factors not entirely included in the CI. This process could also lead to the evaluation of specific aspects of the situation (e.g. informal caregiver exhaustion, pain). Finally, this should allow nurses to analyse the complexity more precisely to discuss interprofessional coordination and specify the care plan. The international nature of the interRAI‐HC and the common structure and coding across instruments allow for deriving a CI from virtually all available versions, hence fostering international dissemination of results and comparisons across different countries.

### 4.3 Limitations

The findings are very encouraging, yet the study suffers from several limitations that need to be addressed. First, all participants volunteered to take part in the study, which is a bias—accounting for lower complexity levels—that cannot be excluded. In this sense, it was observed that the percentage of “yes” responses on the COMID was descriptively lower than that previously found in a clinical population receiving home care (Vallet et al., 2019). Replication of the study with a clinical sample of home care recipients would be necessary to overcome this limit. Doing so would allow for assessing the validity of the proposed algorithm based on clinical situations and everyday complexity as encountered routinely by home care nurses.

Another limitation concerns the quality of the nurses' appraisal of each situation. Indeed, the appraisal required in the fraXity study substantially differs from a routine appraisal where the nurse has become an “expert” of her patients. Although the study protocol fits the clinical situation and possible (i.e. visits at home, the completion of the interRAI‐HC and then the COMID, as is done in clinical contexts), nurses in clinical practice usually have better knowledge of the situation because in addition to assessment, they also provide patient care. Again, replicating the study with a clinical sample, in a routine home care setting, would allow for addressing this drawback.

## 5 CONCLUSION

The study reported here demonstrates that a CI can be derived from the interRAI‐HC, hence tooling up nurses with a means to assess complexity in clinical home care routines. The results can be viewed as a proof of concept, yet they call for replications in larger, clinical samples.

The interRAI‐HC is an instrument used internationally in clinical home care practice. Thus, the CI has an important potential for implementation and for further studies to test it on clinical samples, as well as to assess the psychometric characteristics of the CI, with a special interest in its predictive validity on adverse health outcomes.

The interRAI‐HC is rich enough in its data set to reconsider patient issues and resources through the concept of complexity in a less linear approach. From this, too, it is possible to tool the nurses to identify complex situations. The development of guidelines will enable a generalized understanding and use, positioning the nurse even more as an essential actor in the health system (Busnel et al., 2020).

## CONFLICT OF INTEREST

The authors declare they have no conflicts of interest.

## AUTHOR CONTRIBUTIONS

CB and CL: Study concept and design. CL and CB: Acquisition of data. FV, CB and CL: Analysis and interpretation of data. CB and FV: Drafting of the article. CL: Critical revision of the manuscript. All authors read and approved the final manuscript.

## Supporting information

Table S1‐S2

## Data Availability

The data sets generated (coded, free of personal information), used and analysed during the fraXity study will be deposited at the end of the study at DARIS/FORS (http://forscenter.ch) for data sharing and reuse purposes. FORS/DARIS complies with the FAIR (findable, acceptable, interoperable, reusable) principles.
