# Supplementary material for: Tooling nurses to assess complexity in routine home care practice: Derivation of a complexity index from the interRAI‐HC
Source: Nurs Open. 2020 Nov 25;8(2):815–23. doi: 10.1002/nop2.686 (PMC7877136; doi:10.1002/nop2.686)
Supplement: Supplementary file 1 — Table S1‐S2 [file NOP2-8-815-s001.docx]

**Supplementary material 1.**

**Table presenting the calculation of the CI variables including the description and the coding of the interRAI items, the formula used for combinations and the specificity of coding in fraXity.**

| **COMID n°** | **COMID label** | **Formula to combine interRAI items** | | **interRAI items names** | | **interRAI items coding =1 ^a^** | | **interRAI items description** | | **Specificity of coding in fraXity** ^b^ | | | **Corresponding items in the interRAI-HC Canadian French Version** ^c^ | |  |
| --- | --- | --- | --- | --- | --- | --- | --- | --- | --- | --- | --- | --- | --- | --- | --- |
| 1a | Several chronic diseases (more than 2) and/or unexplained symptoms | sum≥3 | |  | |  | |  | |  | | |  | |  |
|  |  |  | | I1c | | I1c≥1 | | Diagnostic ^d^: Alzheimer’s disease | |  | | |  | |  |
|  |  |  | | I1d | | I1d≥1 | | Diagnostic: Dementia other than Alzheimer’s disease | |  | | |  | |  |
|  |  |  | | I1f | | I1f ≥1 | | Diagnostic: Multiple sclerosis | |  | | |  | |  |
|  |  |  | | I1h | | I1h ≥1 | | Diagnostic: Parkinson’s disease | |  | | |  | |  |
|  |  |  | | I1j | | I1j ≥1 | | Diagnostic: Stroke, cardiovascular accident | |  | | |  | |  |
|  |  |  | | I1k | | I1k ≥1 | | Diagnostic: Coronary heart disease | |  | | |  | |  |
|  |  |  | | I1l | | I1l ≥1 | | Diagnostic: Chronic obstructive pulmonary disease (COPD) | | In fraXity, the item is named I1m | | |  | |  |
|  |  |  | | I1m | | I1m≥1 | | Diagnostic: Congestive heart failure | | In fraXity, the item is named I1l | | |  | |  |
|  |  |  | | I1t | | I1t ≥1 | | Diagnostic: Cancer | |  | | |  | |  |
|  |  |  | | I1u | | I1u ≥1 | | Diagnostic: Diabetes mellitus | |  | | |  | |  |
|  |  |  | | BMI (K1b,K1a) | | (K1b/ ((K1a/100)^2^) ≥ 30 | | Obesity: Body mass index ≥ 30 [BMI = weight in kg/(height in m)^2^] | |  | | |  | |  |
|  |  |  | | J3u | | J3u ≥1 | | Presence of peripheral oedema | |  | | |  | |  |
|  |  |  | | J4 | | J4 ≥1 | | Presence of dyspnea | |  | | |  | |  |
|  |  |  | | I2 | | IF (I2a OR I2b OR I2c OR I2d is not missing) | | At least one other diagnostic reported in the “other diagnostic”questions | |  | | |  | |  |
| 1b | Chronic pain |  | | J6a | | J6a≥1 | | Any pain (any mention on the frequency scale, whether it was manifested or not during the last 3 days) | |  | | |  | |  |
| 1c | Any allergies and/or drug intolerances |  | | M2 | | M2=1 | | A known drug allergy | |  | | |  | |  |
| 1d | Polymedication |  | | M1 | | SUM of medication that are not PRN ≥ 5 | | Five or more substances regularly taken | |  | | |  | |  |
| 1e | Cognitive deficits | C2a=1 AND (C1≥1 or C2b=1 or C2c=1) | |  | |  | |  | |  | | |  | |  |
|  |  |  | | C2a | | C2a=1 | | Short-term memory problem | |  | | |  | |  |
|  |  |  | | C1 | | C1≥1 | | Decision-making regarding tasks of daily life (e.g., when to get up, which clothes to wear or activities to do) not totally independent (i.e., modified independence or minimal or moderate or severe impairment in decision-making) | |  | | |  | |  |
|  |  |  | | C2b | | C2b=1 | | Procedural memory problem | |  | | |  | |  |
|  |  |  | | C2c | | C2c=1 | | Problem in ability to remember | |  | | |  | |  |
| 2a | Financial difficulties and/or an inability to afford the services of assistance, care, treatments, auxiliary devices, a means of transportation, and/or a food supply |  | | Q4 | | Q4=1 | | Financial difficulties | |  | | |  | |  |
| 2b | No informal care, an exhausted informal caregiver, and/or family tensions | ((P1a1=9 and P1a2=9) and P4=0) OR any(P2a, P2b,P2c)=1 | |  | |  | |  | |  | | |  | |  |
|  |  |  | | P1a1, P1a2 | | P1a1=9 and P1a2=9 | | No informal caregiver | |  | | |  | |  |
|  |  |  | | P4 | | P4=0 | | No supportive relationship with family | |  | | |  | |  |
|  |  |  | | P2a | | P2a=1 | | Caregiver unable to continue his/her help | | In fraXity, 2 questions were used for each of the 2 main caregivers. The formula was: (P2aA1=1 or P2aA2=1) | | |  | |  |
|  |  |  | | P2b | | P2b=1 | | Caregiver reports distress or anger | | In fraXity, 2 questions were used for each of the 2 main caregivers. The formula was: (P2bA1=1 or P2bA2=1) | | |  | |  |
|  |  |  | | P2c | | P2c=1 | | Caregiver overwhelmed | |  | | |  | |  |
| 2c | Low level of literacy (related to alphabetization issues, language, and/or cultural barriers) |  | | D2 | | D2≥1 | | Not good and clear understanding | |  | | |  | |  |
| 2d | Social isolation | A13a=1 and ((F1b≠4 and F1c≠4) or F3=2) | |  | |  | |  | |  | | |  | |  |
|  |  |  | | A13a | | A13a=1 | | Living alone | |  | | | A14a | |  |
|  |  |  | | F1b | | F1b≠4 | | Did not receive visits from family and friends during the last 3 days | | In fraXity it is during the last 4 days | | |  | |  |
|  |  |  | | F1c | | F1c≠4 | | Did not have other interactions during the last 3 days | | In fraXity it is during the last 4 days | | |  | |  |
|  |  |  | | F3 | | F3=2 | | Suffering from decline in social activities | |  | | |  | |  |
| 2e | Inadequate housing and/or environmental barriers | Q1a≠0 or Q1b≠0 or Q1e≠0 | |  | |  | |  | |  | | |  | |  |
|  |  |  | | Q1a | | Q1a≠0 | | Degradation | | In fraXity, the modaltity 8="unknown" (not present in the interRAI standard version) was coded complex. | | |  | |  |
|  |  |  | | Q1b | | Q1b≠0 | | Squalid conditions | | In fraXity, the modaltity 8="unknown" (not present in the interRAI standard version) was coded complex. | | |  | |  |
|  |  |  | | Q1e | | Q1e≠0 | | Limited access | | In fraXity, the modaltity 8="unknown" (not present in the interRAI standard version) was coded complex. | | |  | |  |
| 3a | Depression and/or suicidal ideation | I1p≥1 or E2c≥2 | |  | |  | |  | |  | | |  | |  |
|  |  |  | | I1p | | I1p≥1 | | Diagnostic: Depression | |  | | |  | |  |
|  |  |  | | E2c | | E2c≥2 | | Self-reported depressed mood, at least 1 of the last 3 days (or not respond) | |  | | |  | |  |
| 3b | Psychiatric diseases and/or mental disorders (delusions, hallucinations, etc.) | J3g≥1 or J3h≥1 or J3i≥1 or I1q≥1 or I1o≥1 | |  | |  | |  | |  | | |  | |  |
|  |  |  | | J3g | | J3g≥1 | | Abnormal thought process (whether it was manifested or not in the last 3 days) | |  | | |  | |  |
|  |  |  | | J3h | | J3h≥1 | | Delusions (whether it was manifested or not in the last 3 days) | |  | | |  | |  |
|  |  |  | | J3i | | J3i≥1 | | Hallucinations (whether it was manifested or not in the last 3 days) | |  | | |  | |  |
|  |  |  | | I1q | | I1q≥1 | | Diagnostic: Psychosis | |  | | |  | |  |
|  |  |  | | I1o | | I1o≥1 | | Diagnostic: Bipolar disorder | |  | | |  | |  |
| 3c | Addiction | J9a=2 or J9b=3 | |  | |  | |  | |  | | |  | |  |
|  |  |  | | J9a | | J9a=2 | | Daily smoking (including the 3 past days) | |  | | |  | |  |
|  |  |  | | J9b | | J9b=3 | | At least 5 drinks of alcohol in one go during the last 2 weeks | |  | | |  | |  |
| 3d | Anxiety or anguish that renders the clinical picture unclear | I1n≥1 or E2b≥2 | |  | |  | |  | |  | | |  | |  |
|  |  |  | | I1n | | I1n≥1 | | Diagnostic: Anxiety | |  | | |  | |  |
|  |  |  | | E2b | | E2b≥2 | | Self-reported anxious mood, at least 1 of the last 3 days (or not respond) | |  | | |  | |  |
| 3e | Variations in mental function during the day |  | | C3c | | C3c≥1 | | Mental function varies over the day (whether it is usual or not) | |  | | |  | |  |
| 4a | Recurring solicitations of the primary and/or secondary network |  | | E1e | | E1e≥2 | | Repetitive anxious complaints, at least 1 of the last 3 days | | In the interRAI standard version, the complaints are not related to the health although in fraXity the item stated that the complaints are related to the health. | | |  | |  |
| 4b | Ambivalent and/or conflictual communication with a member of the primary and/or secondary network | F1d≥2 and E1b=3 | |  | |  | |  | |  | | |  | |  |
|  |  |  | | F1d | | F1d≥2 | | Conflicts/angry with friends or family in the last 30 days | |  | | |  | |  |
|  |  |  | | E1b | | E1b=3 | | Perpetual anger against oneself or others, each of the 3 last days | |  | | |  | |  |
| 4c | Worries about symptoms, health conditions, and/or medical information |  | | E1d | | E1d≥2 | | Repetitive health complaints, at least 1 of the last 3 days | | |  | | |  | |
| 4d | Aggressiveness (verbal and/or physical) or mutism | E3b≥1 or E3c≥1 | |  | |  | |  | | |  | | |  | |
|  |  |  | | E3b | | E3b≥1 | | Verbal aggressiveness (whether it was manifested or not in the last 3 days) | | |  | | |  | |
|  |  |  | | E3c | | E3c≥1 | | Physical aggressiveness (whether it was manifested or not in the last 3 days) | | |  | | |  | |
| 4e | Resistance or opposition to care, whether active or passive |  | | E3f | | E3f≥1 | | Manifestation of resistance to care (whether it was manifested or not in the last 3 days) | | |  | | |  | |
| 5a | Recent degradation of health status perceived by the patient |  | | J7b | | J7b=1 | | Experiencing an acute crisis or flare-up of a recurrent or chronic problem | | |  | | |  | |
| 5b | Overall change in the degree of independence (ADL/IADL) in the last month | G6≥2 or R2=2 | |  | |  | |  | | |  | | |  | |
|  |  |  | | G6 | | G6≥2 | | Deterioration of the ADL or uncertainty in the performance changes during the last 3 months | | |  | | |  | |
|  |  |  | | R2 | | R2=2 | | A significant change in the general independence for 90 days | | | In the interRAI, the modalities are 0=improved, 1= no change and 2= deteriorated, so the formula is R2=2. In fraXity, the coding being 0=no, 1=yes; the formula was R2=1. | | |  | |
| 5c | Transition period (ex. announcement of diagnosis, hospital discharge, death of caregiver, divorce, work, etc.) | F5=1 or A14≥1 or A13b=1 | |  | |  | |  | | |  | | |  | |
|  |  |  | | F5 | | F5=1 | | Major life stressor during the last 3 months | | |  | | |  | |
|  |  |  | | A14 | | A14≥1 | | Hospitalization less than 90 days ago | | | In fraXity, a modality was added 1= 91 to 180 days ago, so the formula was: A14≥2 | | | A15 | |
|  |  |  | | A13b | | A13b=1 | | Change in the household composition (living with more people) | | | In fraXity, this item can be rated 1 if the person lives with more people but also with fewer people. | | | A14b | |
| 5d | Acute change in cognitive abilities |  | | C5 | | C5≥2 | | Deterioration (or uncertainty) of the decision-taking capacities during the last 3 months | | |  | | |  | |
| 5e | Unpredictability of health status (unusual symptoms, decompensation of a chronic disease, wounds, pain, etc.) | J7a=1 or J7c=1 or C4=1 or N4b≥2 | |  | |  | |  | | |  | | |  | |
|  |  |  | | J7a | | J7a=1 | | Health conditions or diseases making cognitive state, ADL, mood, or behavior patterns unstable | | |  | | |  | |
|  |  |  | | J7c | | J7c=1 | | End-stage disease (6 or fewer months to live) | | |  | | |  | |
|  |  |  | | C4 | | C4=1 | | Acute change of the mental state with regard to the person’s usual functioning | | |  | | |  | |
|  |  |  | | N4b | | N4b≥2 | | At least two emergency room visits during the last 90 days (not counting overnight stay) | | |  | | |  | |
| 6a | Multiple care providers in the secondary network (primary care doctors, medical specialists, formal caregivers, curators, etc.) | | sum≥3 | |  | |  | |  | | |  | |  | |
|  |  | |  | | N3aA | | N3aA≥1 | | Home care assistant: at least 1 day during the last 7 days | | |  | |  | |
|  |  | |  | | N3bA | | N3bA≥1 | | Nurses: at least 1 day during the last 7 days | | |  | |  | |
|  |  | |  | | N3cA | | N3cA≥1 | | Homemaking services: at least 1 day during the last 7 days | | |  | |  | |
|  |  | |  | | N3dA | | N3dA≥1 | | Meal delivery: at least 1 day during the last 7 days | | |  | |  | |
|  |  | |  | | N3eA | | N3eA≥1 | | Physical therapist: at least 1 day during the last 7 days | | |  | |  | |
|  |  | |  | | N3fA | | N3fA≥1 | | Occupational therapist: at least 1 day during the last 7 days | | |  | |  | |
|  |  | |  | | N3gA | | N3gA≥1 | | Speech therapist: at least 1 day during the last 7 days | | |  | |  | |
|  |  | |  | | N3hA | | N3hA≥1 | | Psychological therapist: at least 1 day during the last 7 days | | |  | |  | |
|  |  | |  | | O1 | | O1=1 | | Legal guardian | | |  | | O1a and O1b | |

^a^ In the case where one item from the interRAI-HC was used to create the CI variable, missing data on this interRAI-HC item were also considered missing for the CI variable. In the case where several items from the interRAI-HC were used to create the CI variable, missing data on at least one of the interRAI-HC items were also considered missing for the CI variable, excepted for the 1a. chronic disease item which was coded at missing if less than 10 of the 13 not optional items were completed.

^b^ The adaptation of the coding made for fraXity data presents some differences with the standard version without damaging their comparability. The coding was given for the standard version of the interRAI-HC and the specificity of coding used to analyse the fraXity data were noticed in this column for more clarity.

^c^ The names of items could be different between the standard and the French Canadian versions of the interRAI-HC. When it was the case, the name of the French Canadian version of the interRAI-HC was notified in this column.

^d^ In every diagnosis coded from the items of Section I of the interRAI HC, the diagnosis was coded as present whether the diagnosis was the main disease or not, with or without active treatment

**Supplementary material 2**

**Table. Numbers of answers “yes” for COMID variables and CI variables and and phi testing the link between the CI variable and its corresponding COMID variable, with pairwise deletion method**

| **Short title of the variables** | **COMID** | | | **CI** | | | **Phi between available COMID and CI** |
| --- | --- | --- | --- | --- | --- | --- | --- |
|  | N | No | Yes | N | No | Yes |  |
| 1a. Chronic diseases | 230 | 113 | 117 | 231 | 149 | 82 | 0.51, p<0.001 |
| 1b. Chronic pain | 230 | 74 | 156 | 231 | 62 | 169 | 0.83, p<0.001 |
| 1c. Allergies / drug intolerances | 229 | 170 | 59 | 231 | 168 | 63 | 0.89, p<0.001 |
| 1d. Polymedication | 230 | 135 | 95 | 231 | 131 | 100 | 0.83, p<0.001 |
| 1e. Cognitive deficits | 230 | 210 | 20 | 230 | 177 | 53 | 0.38, p<0.001 |
| 2a.Financial difficulties | 230 | 213 | 17 | 230 | 205 | 25 | 0.62, p<0.001 |
| 2b. Absence or exhaustion of informal caregiver | 230 | 207 | 23 | 230 | 178 | 52 | 0.37, p<0.001 |
| 2c. Low level of literacy | 230 | 220 | 10 | 231 | 211 | 20 | 0.31, p<0.001 |
| 2d. Social isolation | 230 | 204 | 26 | 231 | 198 | 33 | 0.29, p<0.001 |
| 2e. Inadequate housing | 230 | 206 | 24 | 230 | 207 | 23 | 0.41, p<0.001 |
| 3a. Depression and/or suicidal ideation | 230 | 209 | 21 | 230 | 176 | 54 | 0.50, p<0.001 |
| 3b. Psychiatric diseases | 229 | 225 | 4 | 231 | 222 | 9 | 0.32, p<0.001 |
| 3c. Addiction | 230 | 213 | 17 | 231 | 210 | 21 | 0.55, p<0.001 |
| 3d. Anxiety or anguish | 230 | 212 | 18 | 230 | 162 | 68 | 0.38, p<0.001 |
| 3e. Mental function varies over the day | 228 | 220 | 8 | 231 | 225 | 6 | 0.42, p<0.001 |
| 4a. Recurring solicitations | 230 | 219 | 11 | 230 | 200 | 30 | -0.02, p=0.766 |
| 4b. Ambivalent and/or conflictual communication | 229 | 226 | 3 | 230 | 225 | 5 | 0.25, p<0.001 |
| 4c. Worries about symptoms | 230 | 206 | 24 | 230 | 184 | 46 | 0.29, p<0.001 |
| 4d. Aggressiveness | 230 | 227 | 3 | 230 | 226 | 4 | 0.28, p<0.001 |
| 4e. Resistance or opposition to care | 230 | 227 | 3 | 230 | 229 | 1 | 0.58, p<0.001 |
| 5a. Recent degradation of health status perceived by the patient | 230 | 191 | 39 | 231 | 213 | 18 | 0.30, p<0.001 |
| 5b. Change in the ADL/IADL | 230 | 213 | 17 | 229 | 195 | 34 | 0.44, p<0.001 |
| 5c. Transition period | 230 | 205 | 25 | 231 | 154 | 77 | 0.23, p=0.001 |
| 5d. Acute change in cognitive abilities | 230 | 222 | 8 | 231 | 218 | 13 | 0.47, p<0.001 |
| 5e. Unpredictability of health status | 230 | 192 | 38 | 229 | 198 | 31 | 0.45, p<0.001 |
| 6a. Multiple care providers | 230 | 217 | 13 | 231 | 203 | 28 | 0.37, p<0.001 |
| *6b. Absence or low degree of partnership between the different actors* | 230 | 228 | 2 |  |  |  |  |
| *6c. Therapeutic incoherence* | 230 | 230 | 0 |  |  |  |  |
| *6d. Health insurance problems* | 230 | 226 | 4 |  |  |  |  |
| *6e. Emotional and/or physical burden perceived by the secondary network* | 228 | 220 | 8 |  |  |  |  |

Notes. ADL = Activities for Daily Living, IADL = Instrumental Activities for Daily Living
